# Synthesis, Structural Characterization, Hirschfeld Surface Analysis, Density Functional Theory, and Photocatalytic CO_2_ Reduction Activity of a New Ca(II) Complex with a Bis-Schiff Base Ligand

**DOI:** 10.3390/molecules29051047

**Published:** 2024-02-28

**Authors:** Xishi Tai, Xihai Yan, Lihua Wang

**Affiliations:** 1 College of Chemistry and Chemical Engineering, Weifang University, Weifang 261061, China; yan7899@126.com; 2 College of Biology and Oceanography, Weifang University, Weifang 261061, China; wanglihua929@163.com

**Keywords:** 1,3-diamino-2-hydroxypropane, 2-formyl phenoxyacetic acid, Ca(II) complex, synthesis, crystal structure, Hirshfeld surface analysis, photocatalytic CO_2_ reduction

## Abstract

A new bis-Schiff base (L) Ca(II) complex, CaL, was synthesized by the reaction of calcium perchlorate tetrahydrate, 1,3-diamino-2-hydroxypropane, and 2-formyl phenoxyacetic acid in an ethanol–water (v:v = 2:1) solution and characterized by IR, UV-vis, TG-DTA, and X-ray single crystal diffraction analysis. The structural analysis indicates that the Ca(II) complex crystallizes in the monoclinic system, space group *P*12_1_/*n*1, and the Ca(II) ions are six-coordinated with four O atoms (O8, O9, O11, O12, or O1, O2, O4, O6) and two N atoms (N1, N2, or N3, N4) of one bis-Schiff base ligand. The Ca(II) complex forms a tetramer by intermolecular O-H^…^O hydrogen bonds. The tetramer units further form a three-dimensional network structure by π–π stacking interactions of benzene rings. The Hirschfeld surface of the Ca(II) complex shows that the H^…^H contacts represent the largest contribution (41.6%) to the Hirschfeld surface, followed by O^…^H/H^…^O and C^…^H/H^…^C contacts with contributions of 35.1% and 18.1%, respectively. To understand the electronic structure of the Ca(II) complex, the DFT calculations were carried out. The photocatalytic CO_2_ reduction test of the Ca(II) complex exhibited a yield of 47.9 μmol/g (CO) and a CO selectivity of 99.3% after six hours.

## 1. Introduction

With the development of industry, the consumption of fossil fuels is increasingly higher, and their overuse releases large amounts of CO_2_. As is well known, CO_2_ causes a greenhouse effect, environmental pollution, and other problems [1]. Currently, the conversion of CO_2_ into CO, CH_4_, CH_3_OH, HCOOH, etc., by photocatalysis reduction is a promising, new green technology and attracts more and more attention [2,3,4,5,6,7]. So far, many photocatalysts have been found, such as metal-, metal oxide-, metal sulfide-, and carbon-based materials, which show high catalytic activity and selectivity [8,9,10,11,12,13]. However, the shortcomings of expensive costs and difficult synthesis limit the further applications of the above material catalysts. Recently, metal complexes such as cobalt complexes [14,15], Mn tricarbonyl bipyridyl complexes [16], Cu(I) complexes [17], Fe(II)-1,10-phenanthroline complexes [18], Ru(II) binuclear complexes [19], and rare earth metal complexes [20,21] have become a research hotspot in photocatalytic CO_2_ reduction because of their simple synthesis and low price. However, the above studies of complex materials have focused on transition metal and rare earth complexes. The bis-Schiff base metal complexes derived from 1,3-diamino-2-hydroxypropane have attracted much interest because of their coordination diversity [22,23,24]. Calcium (Ca) is the fifth most common element on the earth, and has the advantages of being lightweight, low cost, environmentally friendly, and having a relatively low production cost. Therefore, it is important to synthesize calcium complexes and study their photocatalytic CO_2_ reduction activities.

**Figure 1:**
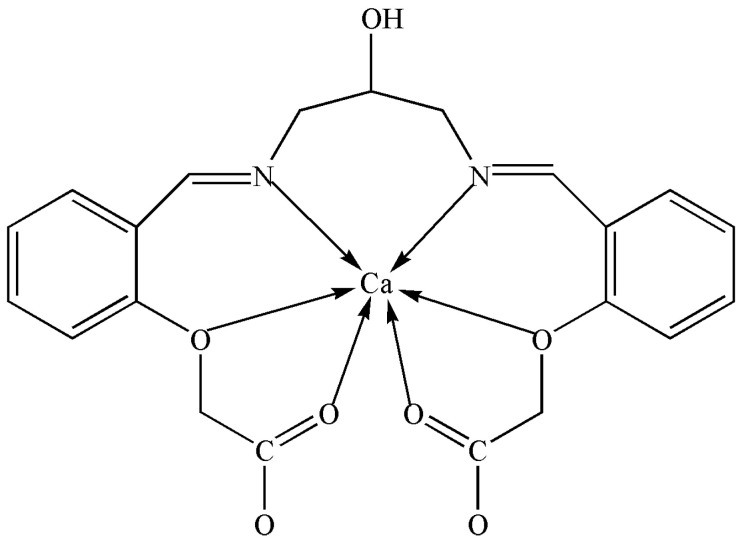
The scheme of the Ca(II) complex.

Herein, we prepare a new bis-Schiff base Ca(II) complex by a one-pot method using calcium perchlorate tetrahydrate, 1,3-diamino-2-hydroxypropane, and 2-formyl phenoxyacetic acid in an ethanol–water solution. The Ca(II) complex was characterized by IR, UV-vis, TG-DTA, and X-ray single crystal diffraction analysis, and the Ca(II) ion was six-coordinated with four O atoms and two N atoms of one bis-Schiff base ligand. Subsequently, the photocatalytic CO_2_ reduction activity of the Ca(II) complex was explored and we found it has excellent catalytic activity with a yield of 47.9 μmol/g (CO) and a CO selectivity of 99.3% after six hours. The Scheme of the Ca(II) complex is shown in Figure 1.

## 2. Results and Discussion

### 2.1. Infrared Spectrum

**Figure 2:**
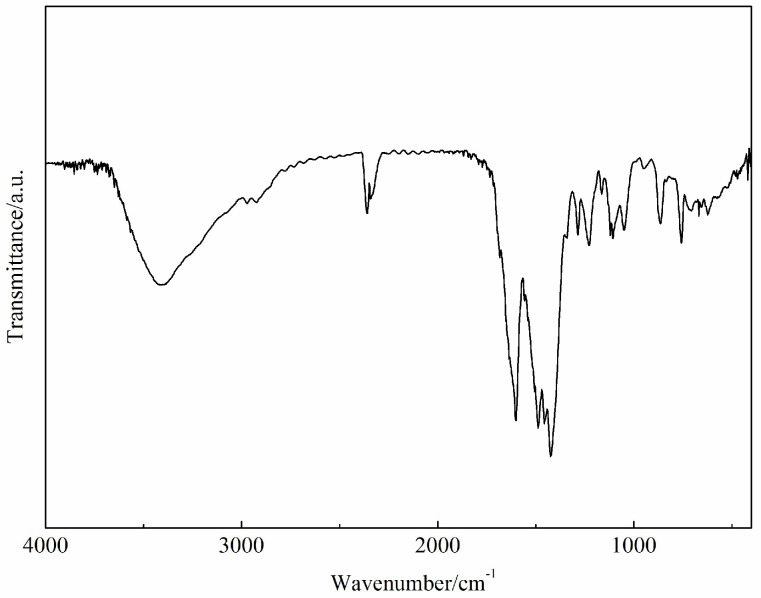
The infrared spectrum of the Ca(II) complex.

The infrared spectrum of the Ca(II) complex is given in Figure 2. In the Ca(II) complex, the characteristic bands of ν_asCOO_^−^, ν_sCOO_^−^, and C=N appeared at ca. 1602, 1425, and 1488 cm^−1^, respectively. This indicates that the O atoms and N atoms of the bis-Schiff base ligand are coordinated with the Ca(II) ion. And the IR results are consistent with the single crystal X-ray structure determination of the Ca(II) complex.

### 2.2. UV-Vis Spectrum

**Figure 3:**
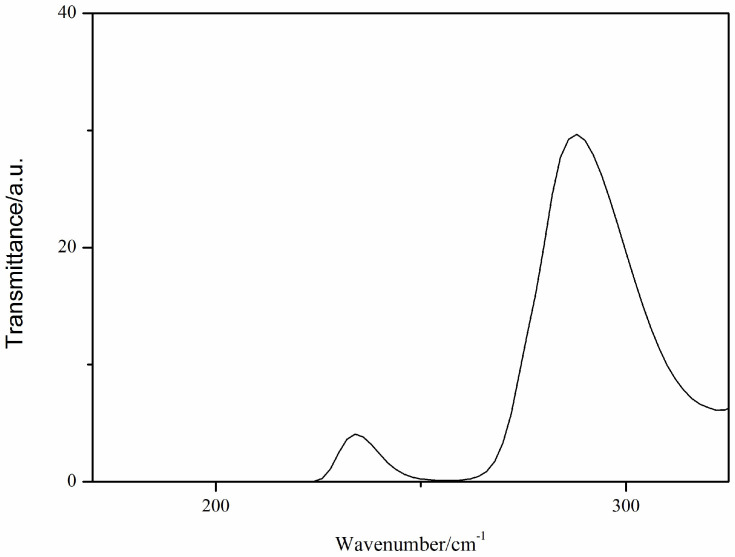
The UV-vis spectrum of the Ca(II) complex.

The spectrum of the Ca(II) complex shows two absorption bands at 234 and 288 cm^−1^ (Figure 3), which may be assigned to the π–π* transitions of the bis-Schiff base ligand.

### 2.3. Thermogravimetric Analysis

**Figure 4:**
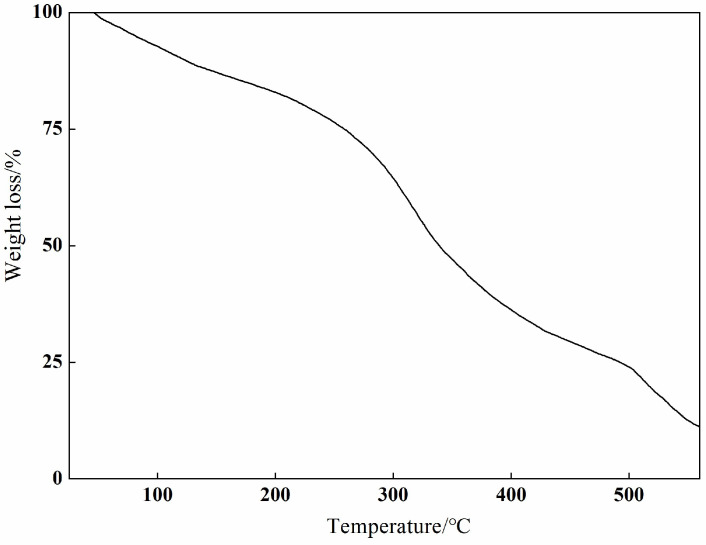
Thermal stability curve of Ca(II) complex.

The thermogravimetric analysis of the Ca(II) complex was determined in an air atmosphere using Al_2_O_3_ as a reference (Figure 4). The first weight loss of 17.09% was observed from 27 to 200 °C, which may be due to the disruption of the intermolecular hydrogen bond of the tetramer and the loss of the adsorbed water molecules. It kept losing weight from 200 to 560 °C, which can be ascribed to the decomposition of the bis-Schiff base (L). The final residue was CaO (found: 11.15%, calculated: 11.69%).

### 2.4. Structural Description of Ca(II) Complex

**Figure 5:**
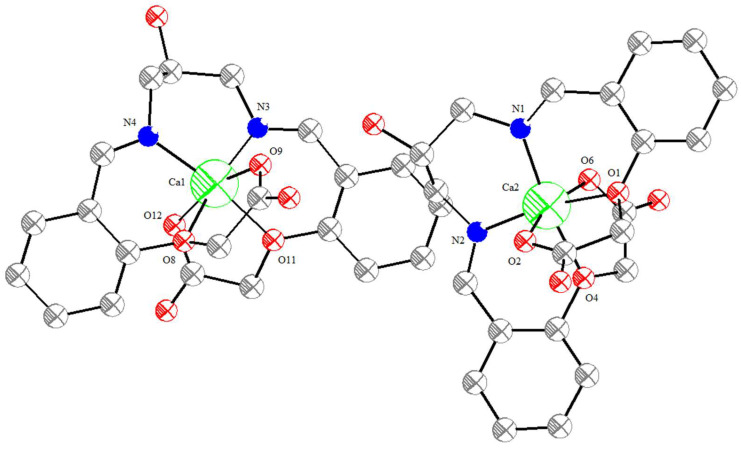
The molecular structure of the Ca(II) complex.

**Figure 6:**
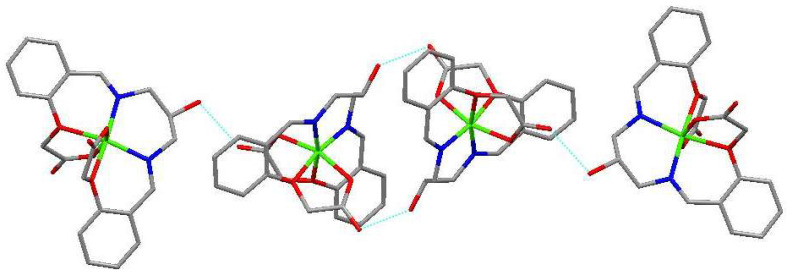
The tetramer formed by intermolecular O-H^…^O hydrogen bonds.

**Figure 7:**
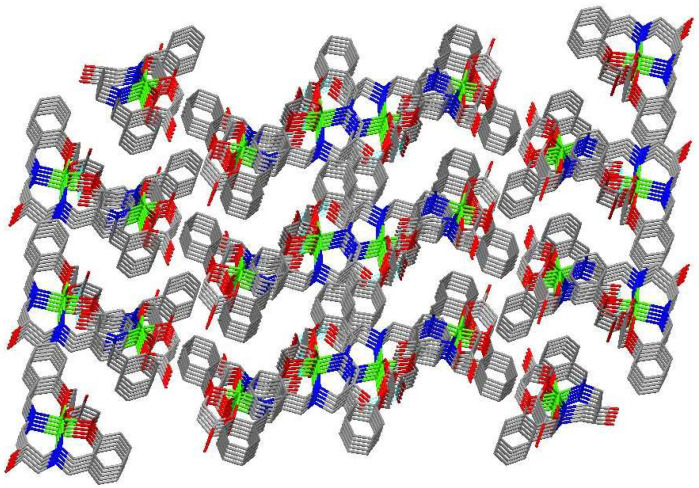
The three-dimensional network structure formed by π–π stacking interactions.

The molecular structure of the Ca(II) complex is shown in Figure 5. Selected bond lengths (Å) and angles (°) for the Ca(II) complex are given in Table 1. The tetramer formed by intermolecular O-H^…^O hydrogen bonds is given in Figure 6. The three-dimensional network structure is shown in Figure 7. The asymmetric unit of the Ca(II) complex contains one Ca(II) ion and one bis-Schiff base ligand (Figure 5). The Ca(II) center is six-coordinated with two imino N atoms (N1 and N2), two ether O atoms (O1 and O4), and two carboxylate O atoms (O2 and O6) of one bis-Schiff base ligand to achieve a distorted octahedral geometry. The bis-Schiff base ligand forms three adjacent six-membered rings and two adjacent five-membered rings with a Ca(II) center, namely ring 1 (N1-Ca2-N2-C10-C9-C8-N1), ring 2 (N1-Ca2-O1-C1-C6-C7-N1), ring 3 (N2-Ca2-O4-C17-C12-C11-N2), ring 4 (O4-Ca2-O6-C19-C18-O4), and ring 5 (O1-Ca2-O2-C21-C20-O1). The dihedral angle of ring 2 and ring 3 is 6.48°, and that of ring 4 and ring 3 is 65.78°, indicating the whole Ca(II) complex molecule is not coplanar. The Ca2-O1, Ca2-O2, Ca2-O4, Ca2-O6, Ca2-N1, and Ca2-N2 distances are 2.089(4), 2.036(4), 2.072(4), 2.031(4), 2.013(4), and 2.024(5) Å, respectively, which are similar to those reported in the literature. The bond angles around the Ca(II) center are 170.52(18)°(N1-Ca2-O4), 93.40(18)° (N2-Ca2-O2), 78.60(15)° (O2-Ca2-O1), 88.30(17)° (O1-Ca2-O6), and 98.18(19)° (O6-Ca2-N2), indicating that N1 and O4 are at the axial place and N2, O2, O1, and O6 are at the equatorial plane. In the crystal, there are two types of O-H^…^O hydrogen bonds: (1) the alcohol oxygen atom is hydrogen-bonded to the alcohol oxygen atom and (2) the alcohol oxygen atom is hydrogen-bonded to the carboxylate O atom. The Ca(II) complex molecules form a tetramer by intermolecular O-H^…^O hydrogen bonds (Figure 6). The tetramer units further form a three-dimensional network structure by π–π stacking interactions of benzene rings (Figure 7). Detailed parameters of the hydrogen bonds in the Ca(II) complex are given in Table 2.

### 2.5. DFT Computation

**Figure 8:**
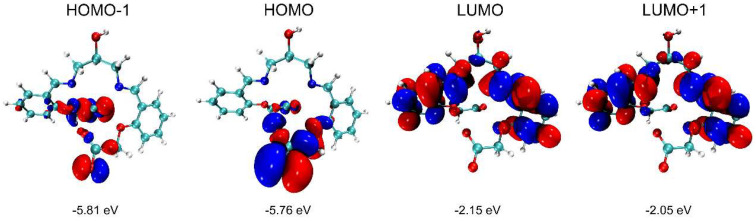
Electron density distributions and energy levels of HOMO−1, HOMO, LUMO, and LUMO+1 for the Ca(II) complex (isovalue = 0.02 e·bohr^−3^).

DFT calculations were carried out to understand the electronic structure of this complex with the Gaussian 16 package [25]. The geometry was optimized at the theoretical level of B3LYP/6-31G* [26,27]. The electron density distributions and energy levels of the frontier molecular orbitals are shown in Figure 8, wherein the visualization was realized using the VMD package 1.9 and the Multiwfn program 3.6 [28]. As shown in Figure 8, HOMO-1 and HOMO are mainly located on each terminal-OCH_2_COO^−^, while LUMO and LUMO+1 are located on the benzene rings and nitrogen atoms on both sides. Compared to the ligand, the coordinated Ca(II) changes the electron density distributions of the frontier molecular orbitals, especially in that the carboxyl groups contribute to the frontier molecular orbitals (Figure S1).

### 2.6. Hirshfeld Surface Analysis of Ca(II) Complex

**Figure 9:**
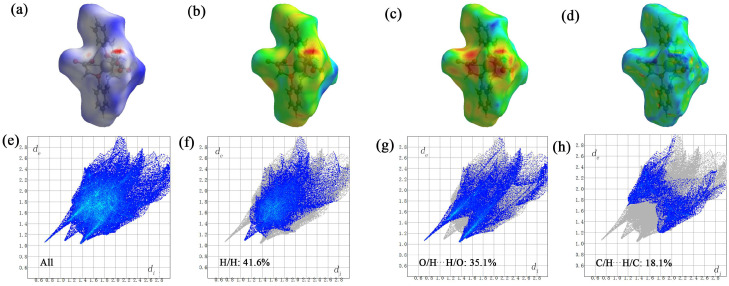
The Hirshfeld surface of the Ca(II) complex. *d*_norm_ (**a**), *d*_i_ (**b**), *d*_e_ (**c**) and curvedness of the crystal (**d**); and the two-dimensional (2D) fingerprint plots representing the overall (**e**) and the top three interactions (H^…^H (**f**), O^…^H/H^…^O (**g**) and C^…^H/H^…^C (**h**)).

The Hirshfeld surface of the Ca(II) complex was analyzed by the CrystalExplorer software 21.5. The Hirshfeld surfaces mapped over *d*_norm_, *d*_i_, and *d*_e_; the curvedness of the crystal (Figure 9a–d); and the two-dimensional (2D) fingerprint plots representing the overall and the top three interactions (H^…^H, O^…^H/H^…^O and C^…^H/H^…^C) are shown in (Figure 9e–h). Based on the calculations, it can be concluded that the H^…^H contacts represented the largest contribution (41.6%) to the Hirshfeld surface, followed by the O^…^H/H^…^O and C^…^H/H^…^C contacts with contributions of 35.1% and 18.1%, respectively. It is worth noting that the pi–pi stacking interactions played a subordinate role in forming the crystal for the C^…^C contacts with a Hirshfeld surface contribution percentage of 3.3%.

### 2.7. Photocatalytic CO_2_ Reduction Activity of Ca(II) Complex

**Figure 10:**
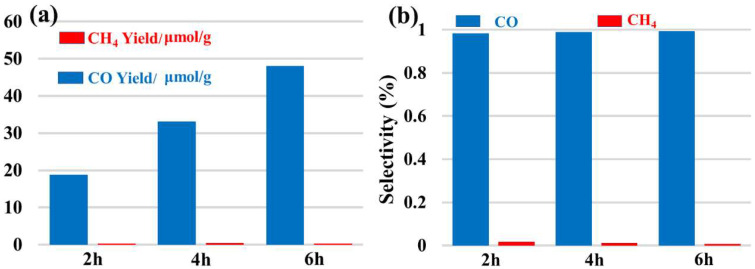
(**a**) Photocatalytic CO_2_ reduction performance and (**b**) product selectivity of Ca(II) complex.

The photocatalytic CO_2_ reduction activity of the Ca(II) complex sample was carried out to explore its application in the CO_2_ reduction field. As shown in Figure 10, the Ca(II) complex sample exhibits obvious catalytic performance. The main product is CO, and the yield gradually increases with the extension of the reaction time, which reaches 47.9 μmol/g after six hours of UV-vis light irradiation. Moreover, the CO selectivity is high; it can achieve a value of 99.3%. Compared to our previous studies, the Ca(II) complex shows a different activity and selectivity in photocatalytic CO_2_ reduction (Table 3). Based on the above results, we can design and synthesize some Ca(II) complexes to optimize the performance of photocatalytic CO_2_ reduction in future studies. In addition, a possible mechanism of CO_2_ reduction over this catalyst is as follows: the Ca(II) complex could be excited to generate electrons and holes under UV-vis light irradiation. The photogenerated electrons could reduce adsorbed CO_2_ molecules to CO gas. Simultaneously, the holes could oxidize water molecules, giving rise to O_2_. On the other hand, the OH group in organic ligands could play an important role in CO_2_ capture capacity.

## 3. Experimental Section

### 3.1. Materials and Measurements

The materials of calcium perchlorate tetrahydrate, 1,3-diamino-2-hydroxypropane, 2-formyl phenoxyacetic acid, and NaOH were used as received from Jilin Chinese Academy of Sciences-Yanshen Technology Co., Ltd. (Jilin, China). IR spectrum was recorded on a Tianjin Gangdong (Tianjin, China) FTIR-850 spectrophotometer (KBr discs, range 4000–400 cm^–1^). UV-vis spectrum was recorded on a PERSEE (Beijing, China) T9 spectrophotometer in the 190–700 nm region in water solution. TG-DTA was performed on a HENVEN (Beijing, China) HCT-2 thermal analyzer. The Hirshfeld surface of the Ca(II) complex was analyzed by CrystalExplorer software [29]. The crystal data of the Ca(II) complex were received on a Bruker (Billerica, MA, USA) CCD area detector (SuperNova, Billerica, MA, USA, Dual, Cu at zero, 296.15 K, multi-scan).

### 3.2. Synthesis of Ca(II) Complex

1,3-Diamino-2-hydroxypropane (0.0901 g, 1.0 mmol), 2-formyl phenoxyacetic acid (0.3602 g, 2.0 mmol), and NaOH (0.080 g, 2.0 mmol) were dissolved in 30 mL ethanol–water (v:v = 2:1) solution with stirring. After 1 h, calcium perchlorate tetrahydrate (0.1606 g, 1.05 mmol) was added to the above solution. The mixed solution was stirred continuously for 5 h at 76 °C and then cooled to room temperature. Colorless needle-like crystals of the Ca(II) complex were obtained after 30 days from filtrate.

### 3.3. Crystal Structure Determination

A colorless single crystal of Ca(II) complex with dimensions of 0.16 mm × 0.12 mm × 0.10 mm was selected for X-ray structure determination. The data were collected on a Bruker Smart CCD diffractometer at 294(2) K using Olex2 [30]. The structure was solved using the SHELXS program [31] and refined with the SHELXL [32] program. Coordinates of hydrogen atoms were refined without any constraints or restraints. All non-hydrogen atoms were refined anisotropically. The hydrogen atoms were positioned geometrically (C-H = 0.95–1.00 Å and O-H = 0.84 Å). Their *U*_iso_ values were set to 1.2 *U*_iso_ or 1.5 *U*_iso_ of the parent atoms. The crystal data and structural parameters of the Ca(II) complex are listed in Table 4.

Crystallographic data for the structure reported in this paper have been deposited with the Cambridge Crystallographic Data Centre as supplementary publication No. CCDC 2307403. The CIF file can be obtained conveniently from the website: https://www.ccdc.cam.ac.uk/structures, accessed on 11 February 2024.

### 3.4. Photocatalytic CO_2_ Reduction Test

Firstly, 100 mL of deionized water and 50 mg Ca(II) complex catalyst were mixed in in a quartz reactor. The high-purity CO_2_ gas was passed into the above suspension and the temperature was kept at 20 °C. After 15 min, we sealed the reactor and turned on a xenon lamp (Beijing Trusttech Co., Ltd., Beijing, China). The gas was analyzed via a gas chromatograph (FID detector, Shandong Huifen Instrument Co., Ltd., Zaozhuang, China, Propark Q column).

## 4. Conclusions

According to the above discussion, a new bis-Schiff base Ca(II) complex was synthesized and demonstrated by IR spectrum, UV-vis spectrum, and X-ray single crystal diffraction analysis. The Hirshfeld surface and DFT calculations of the Ca(II) complex were performed. The photocatalytic CO_2_ reduction activity shows the Ca(II) complex exhibits obvious catalytic performance with a yield of 47.9 μmol/g (CO) and a CO selectivity of 99.3% after six hours. It provides some references for us to continue the study on the synthesis of Ca(II) complexes and their photocatalytic activities of CO_2_ reduction reactions.

## Figures and Tables

**Table 1:** Selected bond lengths (Å) and bond angles (°) for Ca(II) complex.

Bond	*d*	Angle	(°)
Ca1-O8	2.096(4)	O9-Ca1-O8	78.91(14)
Ca1-O9	2.041(4)	O9-Ca1-O11	89.54(17)
Ca1-O11	2.058(4)	O11-Ca1-O8	88.37(16)
Ca1-O12	2.015(4)	O12-Ca1-O8	87.77(16)
Ca1-N3	2.028(5)	O12-Ca1-O9	162.15(17)
Ca1-N4	2.005(4)	O12-Ca1-O11	78.16(15)
Ca2-O1	2.089(4)	O12-Ca1-N3	100.75(18)
Ca2-O2	2.036(4)	N3-Ca1-O8	168.99(16)
Ca2-O4	2.072(4)	N3-Ca1-O9	91.25(17)
Ca2-O6	2.031(4)	O11-Ca1-N3	86.61(17)
Ca2-N1	2.013(4)	N4-Ca1-O8	87.43(16)
Ca2-N2	2.024(5)	N4-Ca1-O9	97.80(17)
		N4-Ca1-O11	170.68(16)
		O12-Ca1-N4	93.37(17)
		N3-Ca1-N4	98.90(17)
		O1-Ca2-O2	78.60(15)
		O2-Ca2-O4	90.64(16)
		O4-Ca2-O1	86.44(16)
		O6-Ca2-O1	88.30(17)
		O6-Ca2-O2	163.44(17)
		O6-Ca2-O4	78.35(16)
		O1-Ca2-N1	89.77(16)
		N1-Ca2-O2	97.13(18)
		N1-Ca2-O4	170.52(18)
		O6-Ca2-N1	92.87(19)
		N1-Ca2-N2	98.36(18)
		N2-Ca2-O1	169.29(18)
		N2-Ca2-O2	93.40(18)
		N2-Ca2-O4	86.54(18)
		N2-Ca2-O6	98.18(19)

**Table 2:** Detailed parameters of hydrogen bonds in Ca(II) complex.

Donor-H	Acceptor	D-H (Å)	H^…^A (Å)	D^…^A (Å)	D-H^…^A (°)
O7-H7	O10	0.84	1.91	2.736(6)	166
O14-H14	O13 ^#1^	0.84	2.00	2.814(7)	162

Symmetric operation code: ^#1^: 1 − x, 1 − y, 1 − z.

**Table 3:** Comparison of activity and selectivity in photocatalytic CO_2_ reduction.

Complex	Yield of CO/μmol/g	CO Selectivity/%
Ca(II) complex	47.9	99.3
Gd(III) complex [21]	22.1	78.5
Yb(III) complex [20]	60.3	100

**Table 4:** The crystal data and structural parameters of the Ca(II) complex.

Empirical formula	C_21_H_20_CaN_2_O_7_
Formula weight	479.49
Temperature/*K*	294(2)
Crystal system	monoclinic
Space group	*P*12_1_/n1
*a*/Å	9.4865(2)
*b*/Å	44.9422(8)
*c*/Å	11.2833(3)
*α*/°	90
*β*/°	114.772(3)
*γ*/°	90
Volume/Å^3^	4367.91(19)
*Z*	8
ρ_calc_, mg/mm^3^	1.458
*μ*/mm^−1^	2.955
*S*	1.070
*F*(000)	2008
Index ranges	−11 ≤ h ≤ 11, −53 ≤ k ≤ 53, −13 ≤ l ≤ 9
Reflections collected	37,707
Independent reflections	7580 [R(int) = 0.0677]
Data/restraints/parameters	7580/0/561
Goodness-of-fit on *F*^2^	1.070
Refinement method	Full-matrix least-squares on *F*^2^
Final *R* indexes [*I* ≥ 2σ (*I*)]	R_1_ = 0.0929, *wR*_2_ = 0.2590
Final *R* indexes [all data]	R_1_ = 0.1016, *wR*_2_ = 0.2692

## Data Availability

Data are contained within the article and Supplementary Materials.

## Supplementary Materials

The following supporting information can be downloaded at: https://www.mdpi.com/article/10.3390/molecules29051047/s1, Figure S1: Electron density distributions and energy levels of HOMO-1, HOMO, LUMO and LUMO+1 for the ligand in (upper) the optimized structure and (below) the adapted crystal structure (isovalue = 0.02 e·bohr^−3^). Table S1: Coordinate of Ca(II) complex.
