# Supplementary material for: Synthesis, Structural Characterization, Hirschfeld Surface Analysis, Density Functional Theory, and Photocatalytic CO2 Reduction Activity of a New Ca(II) Complex with a Bis-Schiff Base Ligand
Source: Molecules. 2024 Feb 28;29(5):1047. doi: 10.3390/molecules29051047 (PMC10934907; doi:10.3390/molecules29051047)
Supplement: Supplementary file 1 [file molecules-29-01047-s001.zip › molecules-2879314-supplementary.pdf]

Synthesis, Structural Characterization, Hirschfeld Surface Analysis, Density Functional Theory, and Photocatalytic CO2 Reduction Activity of a New Ca(II) Complex with a Bis-Schiff Base Ligand

Xi-Shi Tai [1,*], Xi-Hai Yan [1] and Li-Hua Wang [2]

[1] College of Chemistry and Chemical Engineering, Weifang University, Weifang 261061, P. R. China

[2] College of Biology and Oceanography, Weifang University, Weifang 261061, P. R. China

*Correspondence: E-mail:taixs@wfu.edu.cn (X.S. Tai) Telp: +86-536-8785286, Fax: +86-536-8785286

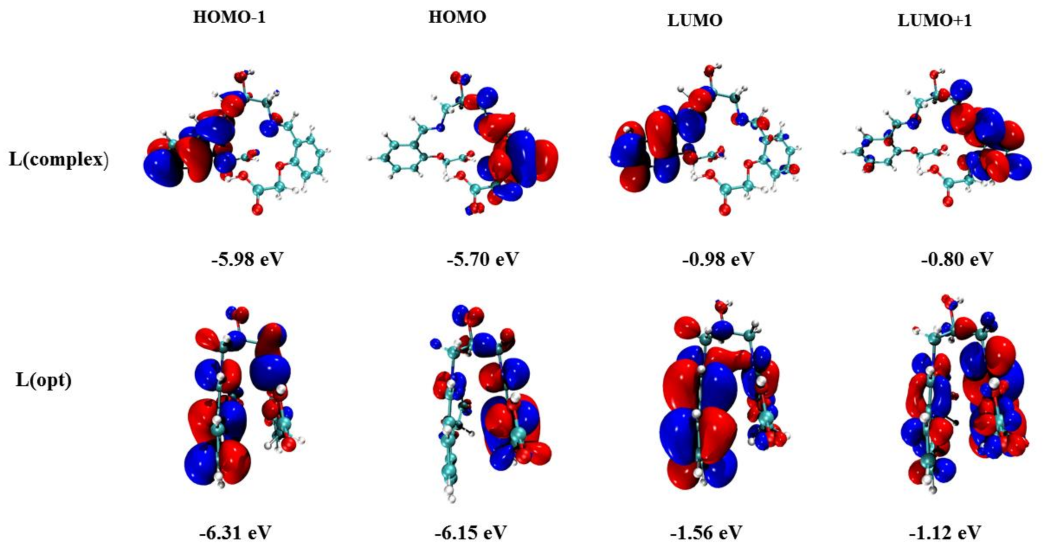

Figure S1. Electron density distributions and energy levels of HOMO-1, HOMO, LUMO and LUMO+1 for the ligand in (upper) the optimized structure and (below) the adapted crystal structure (isovalue = 0.02 e·bohr^{-3}) .

Table S1. Coordinate of Ca(II) complex.

Energy = -2126.057472 hartree

Ca	0.01111400	-0.06310000	0.23077400
O	0.74877300	-2.23995000	3.97390300
O	-0.15315600	-1.07673100	2.24675800
O	-0.49013000	4.98676500	-0.27653900
H	0.08338400	5.37212600	-0.95757500

O	-2.06584000	-0.90282300	-0.94139600
O	0.46456800	-0.85066800	-1.84358500
O	-0.08696800	-2.03618400	-3.69754400
O	2.23873300	-0.78050500	1.08322100
N	-1.78450800	1.69434500	0.40464700
N	1.62626200	1.92667300	0.11790800
C	-3.35820800	-0.87217200	-0.49472500
C	-4.23598000	-1.94240500	-0.70300800
H	-3.89179800	-2.82956400	-1.21888800
C	-5.55005900	-1.88492800	-0.24316500
H	-6.20512100	-2.73539200	-0.40946500
C	-6.02292000	-0.75162400	0.41854200
H	-7.04767300	-0.70134200	0.77195900
C	-5.16002100	0.31813900	0.61012000
H	-5.51835400	1.21266000	1.11347200
C	-3.81840900	0.29008900	0.17880200
C	-3.05514900	1.50997900	0.44352300
H	-3.69590000	2.35546700	0.73085300
C	-1.31492400	3.03782600	0.74928800
H	-0.87842500	3.00518900	1.75722500
H	-2.14273400	3.75881700	0.76990600
C	-0.27082800	3.57766400	-0.25014500
H	-0.46443600	3.13290900	-1.23953600
C	1.19273100	3.32570300	0.16020500
H	1.84146400	3.95058200	-0.47604200
H	1.30768900	3.69301100	1.18946500
C	2.82212600	1.73549000	-0.32050100
H	3.37557400	2.60406800	-0.70679800
C	3.61972100	0.51094800	-0.37586300
C	4.79302000	0.59473000	-1.15296700
H	4.98630500	1.51854700	-1.69258000
C	5.69606100	-0.45396600	-1.24712600
H	6.58416600	-0.36127900	-1.86366100
C	5.44800200	-1.62040200	-0.52278700
H	6.14533100	-2.45204600	-0.56942600
C	4.30856400	-1.73675000	0.27044600
H	4.13943600	-2.64925100	0.82731700
C	3.37959000	-0.69305400	0.33846300
C	-0.31401900	-1.52296000	-2.60753300
C	-1.74419800	-1.78886300	-2.06317300
H	-2.48105800	-1.62925500	-2.85352600
H	-1.79670000	-2.82938000	-1.72494900
C	0.78344100	-1.67751400	2.88578200
C	2.14989000	-1.77440200	2.15100200

H	2.97322200	-1.61178200	2.85027600
H	2.23264200	-2.77762700	1.71898400
